# Bleeding tendency in dual antiplatelet therapy with aspirin/clopidogrel: rescue of the template bleeding time in a single-center prospective study

**DOI:** 10.1186/1477-9560-10-3

**Published:** 2012-01-11

**Authors:** Raul Altman, Ana J Rivas, Claudio D Gonzalez

**Affiliations:** 1 Centro de Trombosis de Buenos Aires, Buenos Aires, Argentina; 2 Department of Pharmacology, School of Medicine, University of Buenos Aires, Buenos Aires, Argentina; 3 Favaloro University, Buenos Aires, Argentina

**Keywords:** nuisance bleeding, bleeding time, platelet, inhibition of platelet aggregation, IPA

## Abstract

**Background:**

Patients with heightened platelet reactivity in response to antiplatelet agents are at an increased risk of recurrent ischemic events. However, there is a lack of diagnostic criteria for increased response to combined aspirin/clopidogrel therapy. The challenge is to identify patients at risk of bleeding. This study sought to characterize bleeding tendency in patients treated with aspirin and clopidogrel.

**Patients/methods:**

In a single-center prospective study, 100 patients under long-term aspirin/clopidogrel treatment, the effect of therapy was assayed by template bleeding time (BT) and the inhibition of platelet aggregation (IPA) by light transmission aggregometry (LTA). Arachidonic acid (0.625 mmol/L) and adenosine diphosphate (ADP; 2, 4, and 8 μmol/L) were used as platelet agonists.

**Results:**

Bleeding episodes (28 nuisance, 2 hematuria [1 severe], 1 severe proctorrhagia, 1 severe epistaxis) were significantly more frequent in patients with longer BT. Template BT ≥ 24 min was associated with bleeding episodes (28 of 32). Risk of bleeding increased 17.4% for each 1 min increase in BT. Correlation was found between BT and IPAmax in response to ADP 2 μmol/L but not to ADP 4 or 8 μmol/L.

**Conclusion:**

In patients treated with dual aspirin/clopidogrel therapy, nuisance and internal bleeding were significantly associated with template BT and with IPAmax in response to ADP 2 μmol/L but not in response to ADP 4 μmol/L or 8 μmol/L.

## Introduction

Until recently, long-term antiplatelet therapy for the prevention of atherothrombotic disease was limited to aspirin (ASA). The availability of thienopyridines, particularly clopidogrel (CLOP), represented an important addition to the physician's armamentarium. The combination of CLOP and ASA in patients with acute coronary syndrome (ACS) reduces the risk of reinfarction, stroke, and death by 20% compared with ASA alone [1]. Nevertheless, the current therapy options for these patients are suboptimal. Despite the use of available antiplatelet therapies, the recurrence of ischemic events in patients with ACS is still increasing and bleeding remains an important, and often underappreciated, risk with these therapies. Patient noncompliance because of bleeding contributes to thrombosis events.

The exact mechanism of benefit has not yet been elucidated but is clearly related not just to inhibition of platelet aggregation (IPA) but also to modification of the many consequences of the platelet activation-endothelial relationships [2]. The current gold standard for testing platelet function is light transmission platelet aggregometry (LTA), which is used to categorize patients receiving ASA and/or CLOP therapy as responders or nonresponders.

Several published studies deal with low platelet responsiveness, the diagnosis of platelet resistance or platelet failure [3] and show that patients with heightened platelet reactivity to antiplatelet agents (ie, low IPA) might be at increased risk of recurrent ischemic events, particularly when they undergo percutaneous coronary intervention [4-6].

But the clear challenge of responsiveness to ASA, CLOP, and other thienopyridine derivatives is to identify the patients at risk of bleeding who will benefit from antiplatelet therapy without hemorrhagic complications. Effectiveness (thrombosis prevention) with adequate safety (no or few hemorrhagic events) is an unresolved clinical problem, and the issue remains constrained by considerable differences between testing methods. A potential association between platelet response and bleeding in patients on combined therapy of aspirin and clopidogrel undergoing coronary stent placement [7] and in other clinical situations [8] has been reported.

This prospective observational study was designed to determine the relationship between template bleeding time (BT) and bleeding complications in patients receiving ASA and CLOP. We also sought to determine if there is a correlation between BT and IPA through LTA studies of platelet-rich plasma (PRP).

## Methods

### Patients

We enrolled 109 patients (67 men, 42 women) who were referred to our Thrombosis Center for thrombophilia screening. Nine patients were excluded at entry (5 men, 4 women): 3 because of a history of superficial or nuisance bleeding, 1 because of von Willebrand disease, 1 because of essential thrombocytemia, 1 with ischemic optical neuritis, 1 with Raynaud syndrome, and 2 with amaurosis fugax, Thus, 100 patients treated with dual antiplatelet therapy, indicated by their own physician (81-100 mg ASA daily and 75 mg CLOP daily), were included in the study. This study was approved by the review board and all participants provided informed consent before being enrolled in the study.

The study participants included 62 men and 38 women, mean age 61.4 (SD ± 11.8) years, median age 62 years, range 35-83 years) with no history of hemorrhagic diseases referred because of previous vascular cerebral symptomatology (1 stroke, 19 transient ischemic events, 17 with ischemic cerebral focus by nuclear magnetic resonance), myocardial infarction or coronary artery bypass graft (*n *= 14), one or multiple coronary stents (*n *= 46, 38 coronary drug-eluting stents), and peripheral arterial disease (*n *= 3).Bleeding was defined according to Ben-Dor et al. [9]: alarming bleeding, internal bleeding, and nuisance bleeding. Internal bleeding included hematoma, epistaxis, vaginal bleeding, melena, hematemesis, eye bleeding, and haematuria. Nuisance bleeding included easy bruising, bleeding from small cuts, petechia, and ecchymosis and was assessed during routine clinical follow-up. Only one bleeding episode was recorded for each patient.

### Additional Medical Therapy

Most patients were receiving additional therapy at the time of the study: antidepressants (*n *= 6), antidiabetics (*n *= 11), anxiolytics (*n *= 26), beta-blockers (*n *= 36), ACE inhibitors (*n *= 23), folic acid (*n *= 11), lipid-lowering agents (*n *= 53), protein pump inhibitor (PPI) (*n *= 16, 7 on omeprazol), levothyroxine (*n *= 7), and diuretics (*n *= 3). Drug-drug interaction between the PPI omeprazole and CLOP that attenuates the antiplatelet effect has been described recently but is not yet widely accepted [10-13]; the US Food and Drug Administration has alerted the public to new safety information concerning an interaction between the 2 therapies. Because of these contradicting publications and, to the best of our knowledge, the absence of any publications showing that PPI drugs affect BT, patients receiving omeprazole therapy were not excluded from the study. None of the patients were receiving anticoagulant therapy.

Patients presented several comorbidities including diabetes (11%), hypertension (57%), and dyslipidemia (53%); 5% had peripheral vascular disease. No patients were active smokers. The mean number of weeks on clopidogrel + aspirin was 28.3 ± 10.6 weeks (max 58 weeks, min 12 weeks).

### Hemostasis Tests and Bleeding Time

Venous blood was drawn from the antecubital vein without stasis and mixed with 0.11 mol/L sodium citrate (1:10 v/v). PRP was obtained by centrifugation at 150 ×*g *for 10 min at room temperature; platelet-poor plasma (PPP) was obtained by centrifugation of PRP at 900 ×*g *for 15 min at 20°C. The PRP was adjusted to a platelet count of 290,000-310,000/μL with autologous PPP. If contamination of the PRP with erythrocytes or leukocytes was observed by light microscopy, a second centrifugation at 900 ×*g *for 5 min was carried out to minimize the cell number. Plastic syringes, tubes, and pipettes were used for all tests. LTA of the PRP was performed in a double-channel Lumi-Aggregometer (Chrono-log Corp., Havertown, PA, USA). Light transmission was set at 10% for PRP and 90% for PPP. The aggregating agent (1-10 μL) was added to PRP in the aggregometer at 37°C with constant stirring (1000 rpm). Arachidonic acid (AA) sodium salt (0.625 mmol/L; Sigma, St. Louis, MO, USA) and adenosine diphosphate (ADP; 2, 4, and 8 μmol/L; Sigma, St. Louis, MO, USA) were used as platelet agonists in the aggregation studies. The percentage IPA was expressed as 100% minus the maximal percent change in light transmission from baseline with PPP used as a reference.

For BT, a disposable Surgicutt adult device (ITC, Thoratec Co, Edison, NJ, USA) was used to make a standard incision (5-mm long, 1-mm deep) on the volar surface of the forearm, perpendicular to the antecubital crease, while maintaining a pressure of 40 mmHg in a sphygmomanometer cuff. The time until complete cessation of bleeding (to the nearest 30 s) was recorded as the BT. Bleeding was followed for a maximum of 26 min. The BT and analysis of platelet function were performed in the morning of the same day.

Basic hemostasis studies, prothrombin time, activated partial prothrombin time, and platelet count were performed at entry. Other tests exploring hemostasis were performed when indicated. Platelet function analyses were performed in accordance with a standardized protocol by the same trained technician, who was not aware of the study objectives or drug intake.

### Statistical Analysis

The nature of the quantitative variable distribution was evaluated by the Shapiro-Wilk test. Differences among groups of quantitative data were explored by ANOVA (Bonferroni post hoc test) or Kruskal-Wallis test in correspondence with the distribution of the variables. Differences among groups in terms of the frequency of patients reporting nuisance bleeding were evaluated by the chi-square test. Correlation between BT and IPA was explored using the Spearman rank order method. The association between nuisance bleeding and quantitative variables, such as BT and IPA, was studied using receiver operating curves (ROC) by obtaining the corresponding area under the curve (AUC) values and their standard errors. Cutoff points were also obtained. The multivariate association between bleeding and BT, (adjusted for gender, age, and IPA) was explored by multiple logistic regression (maximum likelihood; quasi-Newton). Significance was set at *P *< 0.05.

## Results

In our population, the mean BT was 21.5 ± 6.2 min, ranging from 4 min to more than 26 min. The incidence of bleeding was 32%. Fifty-two patients had a BT longer than 26 min: 21 nuisance bleeding, 2 internal bleeding (1 severe hematuria, 1 epistaxis), and 1 alarming bleeding (proctorrhagia with life compromise requiring transfusion). Seventeen patients had a BT longer than 20.5 min and less than 26 min: 5 nuisance bleeding and 1 internal bleeding (mild hematuria) which stopped after discontinuation of platelet inhibitory agents.

The PRP was activated with 0.625 mmol/L AA or 2, 4, or 8 μmol/L ADP. The maximal aggregation at each agonist concentration was taken as the IPA for the calculations.

The IPA resulting from the maximal AA-induced LTA was not different among patients with or without bleeding episodes (Table 1). As expected, there was an inverse correlation between ADP concentration and IPAmax. The Spearman rank order method showed a weak significant correlation between BT and IPAmax (correlation between BT and ADP 2 μmol/L, *r *= 0.32, *P *= 0.001; between BT and ADP 4 μmol/L, *r *= 0.31, *P *= 0.002; between BT and ADP 8 μmol/L, *r *= 0.38, *P *= 0.0002).

**Table 1:** Mean value (± SD) of BT and IPAmax in the study population in response to AA and to different concentrations of ADP

Variable	
Follow-up, weeks, (mean ± SD)	28.3 ± 10.6
Bleeding time, min (mean ± SD)	21.5 ± 6.2
AA 0.625 mmol/L IPAmax %(mean ± SD)	96.2 ± 4.5
ADP 2 μmol/L IPAmax % (mean ± SD)	75.6 ± 13.5
ADP 4 μmol/L IPAmax % (mean ± SD)	69.4 ± 13.4
ADP 8 μmol/L IPAmax % (mean ± SD)	65.0 ± 13.3
Incidence of bleeding (%)	32

In the univariate analysis (Table 2), bleeding events correlated significantly with BT (17% of risk increase per minute of BT, *P *= 0.004) and with ADP 2 μmol/L (4% of risk increase per μmol/L, *P *= 0.049). No correlation was found between bleeding and IPAmax in response to ADP 4 μmol/L or 8 μmol/L. ROC analysis (Table 3) also demonstrated that bleeding events correlated significantly with BT and with IPAmax in response to ADP 2 μmol/L but not in response to ADP 4 μmol/L or 8 μmol/L. According to the ROC, 24 min appears to be the cutoff above which bleeding events are more frequent. In the multivariate logistic analysis, risk for nuisance bleeding was significantly associated with BT (after adjustment for age, gender and ADP2 max values). The multivariate OR was 1.16 per min (95%CI: 1.04-1.30).

**Table 2:** Univariate associations between BT, IPAmax values in response to ADP (independent covariates) and nuisance bleeding (end point): logistic regression

Variables	Odds ratio	95% CI	*P *value
BT (per min)	1.17	1.05-1.31	0.0035
ADP 2 μmol/L IPAmax %	1.04	1.00-1.08	0.0488
ADP 4 μmol/L IPAmax %	1.03	0.99-1.07	0.0876
ADP 8 μmol/L IPAmax %	1.02	0.98-1.06	0.2102

**Table 3:** Association between BT, IPAmax values and nuisance bleeding: ROC AUC

Quantitative variable	ROC AUC	95%CI for AUC	*P *value
BT (min)	0.695	0.595-0.783	0.0009
ADP 2 μmol/L IPA max (%)	0.631	0.529-0.725	0.0330
ADP 4 μmol/L IPA max (%)	0.597	0.495-0.694	0.1170
ADP 8 μmol/L IPA max (%)	0.565	0.462-0.663	0.3016

## Discussion

Besides hyporesponsiveness to CLOP and other thienopyridine derivatives, the clear challenge of antithrombotic therapy is to identify the patients at risk of bleeding who will benefit from antiplatelet therapy without hemorrhagic complications. New data from a study by Shehab et al. [8] showed a substantial risk of bleeding with dual therapy of aspirin plus clopidogrel in a real-world setting.

This study was carried out on patients with vascular pathology or a coronary stent device under prolonged antiplatelets drugs to test the dual therapy effect through LTA and template bleeding time. Ideally, comparing variables measured before and after treatment allows the effect of therapy in each patient to be determined, but comparing BT or LTA at baseline and after treatment with antithrombotic drugs is difficult because patients usually receive treatment very early, before it is possible to perform hemostasis tests. There is no agreement on how responsiveness should be measured concerning devices and cutoff values [14-19].

In the current study, among the 100 patients included in the follow-up, 28 patients had nuisance bleeding, 2 patients had haematuria 1 had proctorrhagia, and 1 patient had epistaxis. Twenty-five patients with nuisance bleeding and the patients with haematuria, epistaxis, and proctorrhagia had longer BT. Twenty-eight of the 32 patients with bleeding episodes had a template BT ≥ 24 min. The univariate analysis and ROC AUC showed that the bleeding events correlated with BT and with ADP 2 μmol/L. In the multiple logistic regression model (risk for nuisance bleeding of BT adjusted by ADP 2 μmol/L, IPAmax (% values)) significant correlation between BT and bleeding events was found (odds ratio (OR) per minute, 1.16 (95% confidence interval (CI) 1.04-1.30, *P *= 0.007). Moreover ≥ 24 min appears to be the cutoff above which nuisance bleeding or severe bleeding is more frequent.

The BTs were compared with regard to platelet aggregation. The IPA is derived from the maximal aggregation obtained in the LTA of PRP. The IPAmax in AA-activated PRP and ADP 4 and 8 μmol/L did not discriminate patients with bleeding tendency. The IPAmax in response to ADP 2 μmol/L does seem to be sensitive for predicting bleeding episodes in patients receiving ASA plus CLOP treatment.

The BT, as well as LTA, is considered to be an inaccurate and poorly reproducible technique, which is dependent on several variables. Therefore, these methods could be inappropriate for measuring platelet inhibition activity [20,21]. Nevertheless, LTA is the gold standard test of platelet function and is used to categorize patients receiving ASA, CLOP, or dual therapy as responders or nonresponders or to define drug resistance. In addition, a recent paper used LTA to compare IPA between patients receiving ticagrelor or CLOP therapy [22]. Antonino et al. [23] found a strong correlation (*P *≤ 0.04) between LTA and flow cytometric measurements. Gremmel et al. [24] found that the results from 4 different assays of platelet function significantly correlated with LTA, and Paniccia et al. [25] found a significant correlation between LTA and VerifyNow but not the PFA-100 assay. Recently, Bonello et al. [26] provided a consensus opinion on the definition of high on-treatment platelet reactivity to ADP based on various methods reported in the literature and proposed LTA as 1 of the 4 tests associated with clinical risk. Very recently, Parodi et al. [5] found that high residual platelet reactivity assessed by LTA and ADP as agonist among patients receiving clopidogrel after percutaneous coronary intervention (PCI) has been associated with a high risk of ischemic events at short- and long-term follow-up.

Breet et al. [20] reported that LTA and 5 other assay methods available for platelet studies were unable to predict which patients were at higher risk of bleeding following stent implantation. In contrast, Tsukahara et al. [27] found that high platelet responsiveness to CLOP or ticlopidine plus ASA, using LTA and ADP as an agonist, is associated with an increased risk of bleeding. Serebruany et al. [28] found that inhibition of platelet aggregation > 50% using 5 μM ADP-induced IPA strongly correlates with minor but not severe bleeding events in a large cohort of patients with coronary artery disease and ischemic stroke treated with chronic low-dose aspirin plus clopidogrel.

Cuiset et al. [29] identified patients post-treatment as hyper-responders when ADP-induced platelet aggregation was below < 40% (IPA > 60%). The risk of TIMI major and minor bleeding was significantly higher in these patients.

None of these studies, although they include a large number of patients, deals with BT, the only in vivo test that detects the relationship between vascular endothelium and platelets.

Template BT is an invasive technique displaying low sensitivity to mild or moderate abnormalities of inherited platelet defects. The situation may be different when the effects of drugs are being tested. According to our results, when BT is carried out by experienced personnel, it is a useful technique for evaluating the in vivo effects of antiplatelet agents. Although a number of other platelet function tests have been developed subsequent to BT [30], these tests have shown poor correlation and agreement between them, and their clinical usefulness for correctly classifying patients remains undetermined [31,32].

In some circumstances, the cessation of ASA/CLOP treatment (eg, a drug-eluting stent in patients undergoing PCI) is a strong risk factor for thrombosis. Several published studies have shown that patients exhibiting heightened platelet reactivity (ie, low IPA) to antiplatelet agents might be at increased risk of recurrent ischemic events, particularly when they undergo PCI [33-35]. Nevertheless, prediction of bleeding in individual patients remains a challenge.

The important question is whether an antiplatelet agent has the desired effectiveness (ie, thrombosis prevention) with adequate safety (ie, few or no hemorrhagic events). What becomes a problem for the patients is even minor or moderate bleeding, as shown by Rao et al. [36] who pooled the data from 4 multicenter, randomized clinical trials of patients with ACS.

The GUSTO bleeding classification was found to identify patients at risk of short- and long-term death and myocardial infarction. Even though Aronow et al. [37] did not find a significant increase in the overall rate of major or minor thrombolysis in myocardial infarction TIMI score bleeding in patients after PCI when adding CLOP to ASA, major gastrointestinal bleeding increased in the year after PCI. Nuisance bleeding is commonly encountered in patients taking dual antiplatelet therapy, and its incidence and impact are important for compliance. Roy et al. [38] found that 32.4% reported bleeding events, of which 85.7% were nuisance, 13.6% were internal, and 0.7% were alarming. In the nuisance bleeding group, the rate of CLOP discontinuation was 11.1%.

According to our results in the present study, template BT indicates which patients are prone to bleeding with combined ASA/CLOP therapy, and there is correlation between BT and IPAmax in response to low concentration of ADP (2 μmol/L) but not at higher concentrations (4 or 8 μmol/L). This finding is not surprising because BT is indicative of the platelet-endothelial cell interactions and platelet aggregation is indicative of platelet-platelet interactions

The initial step in primary hemostasis is the adhesion of platelets to the subendothelial matrix through specific adhesive glycoproteins. The deposition of platelets on the subendothelium involves the platelet receptor GP Ib/IX/V complex, which interacts with the high molecular weight multimeric plasma protein von Willebrand factor. GPIIb/IIIa (integrin α_IIb_β_3_), which links activated platelets through fibrinogen bridges, is the central platelet receptor in aggregation supporting platelet-platelet interactions. The stimulation of platelets as a result of adhesion leads to a spreading activation of GPIIb/IIIa, enabling the binding of soluble fibrinogen, leading to platelet aggregation and granule secretion [39]. Although interconnected, platelet adhesion and aggregation are different steps in hemostasis, and drugs used therapeutically as antithrombotic agents could affect platelet adhesion, platelet aggregation, or both. Aspirin acts by irreversibly acetylating platelet cyclooxygenase-1, thereby blocking the formation of thromboxane A_2_. Clopidogrel acts by blocking the P_2_Y_12 _platelet ADP receptor, thereby inhibiting ADP-induced platelet activation and aggregation. The IPA is probably not the exact mechanism of the benefit of ASA and CLOP because of the effect on the many consequences of platelet activation-endothelial relationships [40].

As a result of combining ASA and CLOP, template BT was prolonged (≥ 26 min) in 55 of 100 patients (55%); it is pretty clear that this antiplatelet combination affects not only platelet aggregation and thrombin generation [41] but also platelet-endothelial cell interactions, modifying primary hemostasis. This modification could be an important, perhaps the most important, effect for thrombosis prevention. Increasing evidence indicates that platelet adhesion is involved in the earliest development of atherosclerotic lesions [42].

In conclusion, although platelet hyporesponsiveness to CLOP has been associated with major adverse cardiovascular events and stent thrombosis, the risk of increased platelet inhibition is bleeding, which has been strongly linked to mortality [43]. We found that, in patients taking ASA plus CLOP who had a prolonged template BT, bleeding events were frequent. Template BT determined by a trained technician could be useful for following patients treated with dual antiplatelet therapy. Among these patients, those with BT ≥ 24 min presented with increased bleeding episodes (nuisance bleeding, epistaxis, hematuria, and proctorrhagia). Moreover, we found correlation between bleeding events and IPAmax in response to feeble concentrations of ADP 2 μmol/L in LTA, consistent with the hypothesis that lower IPA in response to ADP is associated with increased bleeding risk, but definitive studies are necessary to determine the cutoff point of reactivity to ADP associated with bleeding risk. The present results may have greater relevance with the emergence of more potent antiplatelet drugs [44].

The main limitation of our results is the sample size but the statistical differences in BT between patients with no bleeding and patients with bleeding risk support our findings; a larger study is indicated.

## Competing interests

The authors declare that they have no competing interests.

## Authors' contributions

RA designed the study and coordination, drafted the manuscript and discussed the results. AJR carried out the lab assays and discussed the results. DCG performed the statistical analysis and discussed the results and participated in its design. All authors read and approved the final manuscript.
